# Letter from Wm. O Kulp, D.D.S.

**Published:** 1869-04

**Authors:** Wm. O. Kulp

**Affiliations:** Muscatine, Iowa,


### CORRESPONDENCE.

### ARTICLE VI.

*Dear Journal:—*

Your February No. contains another editorial on a " Southern Dental Association," to which I wish to respond, and not from a "*selfish*" point of view either.

I think we in the West and Southwest feel differently towards each other, than those of our Eastern and Southern brethren. We haven't so many rival Colleges to sustain. I have no objection to the formation of a Southern Dental Association, if that Association is not to take the *place* of the *American Dental Association.* I think we ought to sustain but one *National* Association, all others ought to be subject to that, i e send their delegates &c. I think there ought to be more associational effort among our Southern Dentists, (excepting Kentucky and Tennessee which are doing well,) it would have a tendency to make them more harmonious and willing to overlook all things else, for the sake of our great and beloved profession.

We of the West say to our members in the South, organize all the Dental associations you please, only pray! don't convey the impression that your society is to take the place of the American Dental Association to *you*, for we want to meet you as members of the same society yearly, and we want our profession to be free from *all* sectional distinctions.

We have given up our beloved "Western Dental Society" and merged it into State and district societies, and send our delegates annually to our National association, and it has proved a good change, for it has made us larger hearted. We want to see the American Dental Association appoint their next meeting on the banks of the Mississippi River at St. Louis or Memphis, and then we shall feel much better if we know there does not exist a society South which has for its object the snubbing of the old mother of our profession in this country, viz. the A.D.A.

Very truly and fraternally yours,

WM. O. KULP. D.D.S.

*Muscatine, Iowa, February 23rd* 1869.

---

